# Evaluation of Reducing NO and SO_2_ Concentration in Nano SiO_2_-TiO_2_ Photocatalytic Concrete Blocks

**DOI:** 10.3390/ma14237182

**Published:** 2021-11-25

**Authors:** Jong Won Lee, Sang Hyuk Lee, Young Il Jang, Hee Mun Park

**Affiliations:** 1 Department of Highway & Transportation Research, Korea Institute of Civil Engineering and Building Technology, 283, Goyang-daero, Ilsanseo-gu Goyang-si 10223, Korea; asca28@kict.re.kr (J.W.L.); slee@kict.re.kr (S.H.L.); 2 Department of Construction Engineering Education, Chungnam National University, 99 Daehak-ro Yuseong-gu, Daejeon 34134, Korea; jang1001@cnu.ac.kr

**Keywords:** titanium dioxide, photocatalyst, nitrogen oxide, concrete block

## Abstract

The use of titanium dioxide in concrete block pavements is a promising approach to reduce air pollution in the roadside. When TiO_2_ is used as an additive of cement concrete or mortar, it is not dispersed uniformly due to agglomeration between particles causing the degradation of photocatalytic reaction. To improve the photocatalytic performance of TiO_2_, the Nano SiO_2_-TiO_2_ (NST) has been developed by coating TiO_2_ with SiO_2_ as a support using the sol-gel method. The environmental performance of concrete blocks incorporating NST as an additive was evaluated using both laboratory and full-scale chamber experiments. It was observed from laboratory environment chamber testing that the NO reduction efficiency of concrete blocks with 4% NST ranged from 16.5 to 59.1%, depending on the UV intensity. Results of the full-scale chamber test on NST concrete blocks indicated that the NO and SO_2_ reduction efficiencies were 22.3% and 14.4% at a 564 W/m^2^ of solar radiation, respectively. It was found that the increase in UV intensity and solar radiation had a positive effect on decreasing NO and SO_2_ concentration. In the future, the NST will be applied at in-service photocatalytic block pavements to validate the environmental performance in field conditions.

## 1. Introduction

With economic development led by rapid industrial growth, the air pollution has been steadily increasing, due to soot and sulfur oxides from factories and vehicle exhaust emissions, and it has become a rising common interest around the world [1,2]. As for the primary air pollutant NO_x_, road and non-road mobile pollutant sources account for the most proportion at 59%. NO_x_ emitted from road mobile sources such as vehicles and stationary sources including industrial factories power generation facilities is a harmful air pollutant; NO emitted from vehicles is especially a serious problem in metropolitan areas [3,4,5]. In this regard, it is necessary to develop measures to reduce the NO_x_, including NO and NO_2_, from on-road mobile sources for the entire society.

The titanium dioxide (TiO_2_), one of the photocatalytic compounds, has various strengths, including physical and chemical stability, excellent acid resistance, alkali resistance, UV protection, dispersibility, durability, and high reactivity and activity [6]. TiO_2_ absorbs ultraviolet rays with a wavelength of 400 nm or less and is separated into high-energy electrons (e^−^) and holes (h^+^), which react with surface adsorbed oxygen and water to form active species, such as superoxide anions (O_2_^−^) and hydroxyl radicals (OH^−^), respectively. Due to the strong oxidizing power of the active species, TiO_2_ can decompose air pollutants, have a sterilization effect, and remove and absorb harmful gases [7]. It is expected that concrete products with excellent performance can be produced by mixing TiO_2_ with mortar and concrete. When it comes to road structures, it will be very effective in preventing air pollution, as TiO_2_ directly adsorbs and removes harmful gases emitted from vehicles. Its very large, specific surface area will maximize photocatalytic efficiency [8,9,10,11]. When the concrete material with TiO_2_ is applied to the road facilities, it will be very effective in reducing air pollution by directly absorbing and removing the harmful gases from vehicle emissions [12,13]. For this reason, Japan, Hong Kong, and some European countries use TiO_2_ for road structures and concrete blocks in removing air pollutants. In Italy, when photocatalytic (TiO_2_) cement was applied to the concrete block pavement of Borgo Palazzo Street in Bergamo, air pollution in the area was reduced by 30–40%. In Belgium, TiO_2_ block pavement was constructed over an area of 10,000 m^2^ in the Antwerp area, and air pollution decreased by about 20% one year after the construction. In Japan, TiO_2_ concrete block pavement was applied on an area of 50,000 m^2^ in Osaka, Chiba, Chigasaki, and Saitama-Shintoshin. As a result of the study, it was found that the photocatalytic pavements can remove 15% of NO_x_ emitted from vehicles in motion and they have a higher NO_x_ decomposition effect than roadside trees [12,14]. The use of higher content TiO_2_ in concrete material can interrupt the hydration reaction of cement, thus degrading the concrete’s strength [4]. When the TiO_2_ particles inside the concrete is not exposed to light sources or exhaust gases, it is difficult to trigger a photocatalytic reaction [15,16]. In particular, TiO_2_ of nano size with a large specific surface area was not dispersed due to agglomeration between particles, but rather was present as aggregates on the surface of the concrete block, thereby reducing the photocatalytic reaction [17].

In recent years, the nanotechnology has been dramatically developed through the continuous improvements in the production and characterization of solid materials [18,19,20,21,22]. The performance of solid materials is greatly affected by its surface and electronic properties when size is reduced to the nanoscale [23,24,25,26]. In particular, the aggregates are easily formed since nanomaterials have a large specific surface area and great unsaturated bonds when its size gets smaller [27,28]. Therefore, nano-sized TiO_2_ materials could not produce a sufficient effect with mortar and concrete as it exists as aggregates.

In order to improve the aggregation of nano-sized TiO_2_ in the binder, the nano-sized TiO_2_ interface should be deformed. Coating SiO_2_ as a support can create Ti-O-Si bonds on the nano TiO_2_ interface [29,30]. This type of chemical bonding can bring more negative charges to the surface of TiO_2_, which helps dispersion in water-based bonds on the electrostatic repulsion reaction. Accordingly, the dispersing force in the binder can be improved by decreasing the reduction of agglomerates. In addition, adding SiO_2_ as a support to cement is expected to create a pozzolanic reaction, improving concrete properties [31,32,33,34,35].

In order to solve the problems mentioned above, the SiO_2_-TiO_2_ material has been developed by coating TiO_2_ with SiO_2_ as a support using the sol-gel method. The developed Nano SiO_2_-TiO_2_ material was used to fabricate the concrete blocks. The environmental performance of the concrete blocks was evaluated based on the ability to remove air pollutants using laboratory and full-scale environmental chamber experiments.

## 2. Background

### Photocatalyst

The utilization of TiO_2_ on pavement and road facilities has a significant effect on reducing NO, a primary source of fine particulate matter [36,37,38,39,40]. NO is considered the primary pollutant, which is mainly introduced into the atmosphere directly from high-temperature combustion in transport and industrial activities, whereas NO_2_ is considered a secondary pollutant, since it is mostly formed in the atmosphere due to the interaction between NO with O_2_ or O_3_ and/or sunlight. Photocatalysts are capable of decomposing various numbers of oxides and organic compound pollutants that cause health and environmental problems. The governing decomposition mechanism involves the generation of radicals due to the irradiation to the photocatalyst substance, and subsequently, converting pollutants into harmless compounds [36]. 

The photocatalytic process of TiO_2_ is described in nine reactions [41]. This process begins with irradiating UV light to the TiO_2_. When the TiO_2_ absorbs an equal or higher energy than the band gap, an electron is transferred from the valence band to the conduction band. The band gap energy of TiO_2_ in its anatase phase, which is mainly used as a photocatalyst, is 3.2 eV. (Equation (1)).

In this reaction, holes (h^+^), which has great reducing power, reacts with water (H_2_O) to generate hydroxyl radical (OH∙) (Equation (2)), which also presents high oxidizing power. Meanwhile, electrons (e^−^) accomplish the reduction of oxygen (O_2_) molecules to produce superoxide anion (O_2_^−^), which is very effective on pollutant’ degradation (Equation (3)). Thus the O_2_^−^ produced reacts with H^+^ to dissociate in water to form HO_2_∙. (Equation (4)).

(1) $${\mathrm{TiO}}_{2}\;\overset{\mathrm{UV}}{\to }{\;h}^{+}\;+\;{\;e}^{-}$$

(2) $${\;h}^{+}\;+\;{\mathrm{OH}}^{-}\to \;\mathrm{OH}\;\cdot$$

(3) $${\;e}^{-}\;+\;O_{2}\;+\;\to \;{\;O}_{2}^{-}$$

(4) $$O_{2}^{-}\;+\;{\;H}^{+}\;\to \;{\;\mathrm{HO}}_{2}\cdot$$

The mechanism by which O_2_^−^ and OH^−^ produced by the above reaction react with NO_x_ and are removed is as follows.

(5) $$\mathrm{NO}\;+\;\mathrm{OH}\cdot \to \;{\mathrm{HNO}}_{2}$$

(6) $${\mathrm{HNO}}_{2}\;+\;\mathrm{OH}\cdot \to \;{\mathrm{NO}}_{2}\;+\;H_{2}O$$

(7) $$\mathrm{NO}\;+\;{\mathrm{HO}}_{2}\cdot \to \;{\mathrm{NO}}_{2}\;+\;\mathrm{OH}\cdot$$

(8) $${\mathrm{NO}}_{2}\;+\;\mathrm{OH}\cdot \to \;{\mathrm{HNO}}_{3}\cdot$$

(9) $${\mathrm{NO}}_{x}\;+\;O_{2}^{-}\;\to \;{\mathrm{NO}}_{3}^{-}$$

According to Equations (5) and (6), OH∙ produced by the photocatalytic reaction reacts with NO_x_, NO, NO_2_, etc. to finally produce HNO_3_. The HNO_3_ is water-soluble and can be easily removed from the photocatalyst surface by an external environment, such as rain. The O_2_^−^ generates HO_2_∙ by the reaction of Equation (4), and HO_2_ reacts with NO according to Equations (7) and (8) to finally produce HNO_3_.

In addition, in the NO_x_ environment, as shown in Equation (9), NO_x_ molecules react with O_2_^−^ to generate NO_3_^−^, which effectively affects the removal of nitrogen oxides [42]. Similarly, purification of SO_2_ is as follows:

(10) $${\mathrm{SO}}_{2}\;+\;\mathrm{OH}\cdot \;\to \;{\mathrm{HSO}}_{3}$$

(11) $${\mathrm{HSO}}_{3}\;+\;O_{2}\;\to \;{\mathrm{SO}}_{3}$$

(12) $${\mathrm{SO}}_{3}\;+\;H_{2}O\;\to \;H_{2}{\mathrm{SO}}_{4}$$

As shown in Equations (10)–(12), sulfuric acid (H_2_SO_4_) production from SO_2_ oxidation proceeds through a series of radical reactions. The HSO_3_ radical then rapidly reacts with molecular oxygen (O_2_) to yield SO3. SO_3_ reacts with atmospheric moisture (H_2_O) to form H_2_SO_4_. The finally produced H_2_SO_4_ can be easily removed from the photocatalytic surface by external environments, such as rain.

## 3. Materials and Experiment Method

### 3.1. Materials

#### 3.1.1. Nano SiO_2_-TiO_2_

In order to improve the photocatalysis efficiency of TiO_2_, the TiO_2_ material had been developed as an anatase crystal phase with a larger specific surface area and excellent photodissociation. Various attempts had been made, including sputtering, which was a process in which TiO_2_ was directly and physically coated on a proper solid support. Although the specific surface area of TiO_2_ was large, it was difficult to achieve a proper photocatalytic effect due to aggregation of nano-sized particles in the mixture with a binder. To solve these problems, the Nano SiO_2_-TiO_2_ (NST) had been developed using the Sol-gel method in this study.

**Figure 1:**
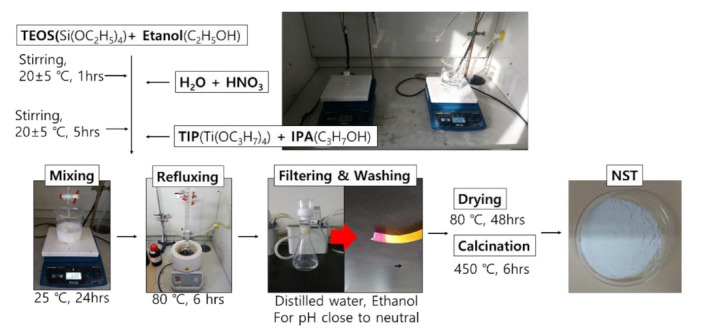
Production process of NST [43].

As for starting materials, TTIP [Titanium Isopropoxide; Ti(OC_3_H_7_)_4_] (Sigma-Aldrich, Seoul, Korea) was used as the precursor of TiO_2_; TEOS [Tetraethyl Orthosilicate; Si(OC_2_H_5_)_4_] (Sigma-Aldrich, Seoul, Korea) as the precursor of SiO_2_; nitric acid (HNO_3_) (Sigma-Aldrich, Seoul, Korea) as the catalyst; and ethanol (EtOH) and iso-propanol (IPA) as the solvent (Sigma-Aldrich, Seoul, Korea). The NST was processed as shown in Figure 1, and the physical properties of the NST were shown in Table 1.

1. TEOS and EtOH were stirred at a molar ratio of 1:1, at 600 rpm or higher, for one hour.
2. HNO_3_ and distilled water were stirred at a molar ratio of 1:150, at 600 rpm or higher, for one hour.
3. The solutions prepared in steps 1 and 2 were stirred at 600 rpm or higher for five hours.
4. TIP and IPA were stirred at a molar ratio of 1:1 at 600 rpm or higher for one hour.
5. The solutions prepared in steps 3 and 4 were mixed and stirred at 800 rpm for 24 hours.
6. The mixture prepared in step 5 was refluxed at 80 °C for six hours.
7. The prepared NST slurry was washed and filtered for neutralization.
8. The NST slurry was dried at 80 °C for 48 hours.
9. Heat treatment was applied to the dried NST at 450 °C for six hours.
10. The heat-treated NST was ground.

**Figure 2:**
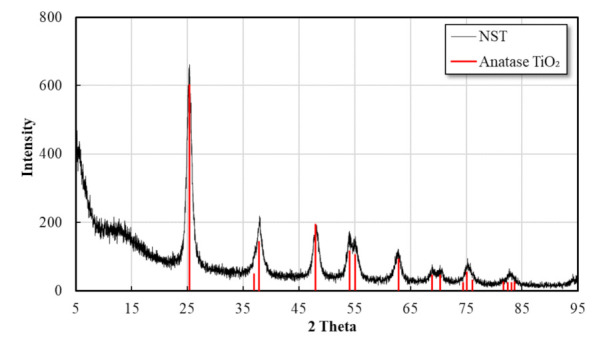
XRD pattern of NST.

**Figure 3:**
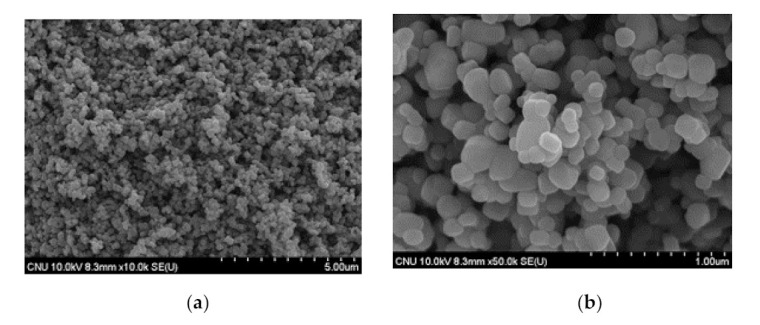
SEM images of NST: (**a**) NST (10,000×); (**b**) NST (50,000×).

**Figure 4:**
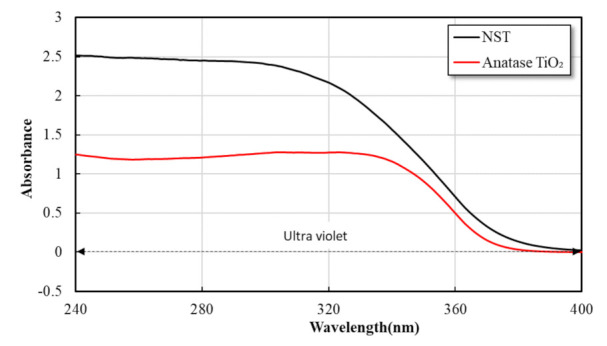
UV-Vis absorbance of NST.

The main performance of NST developed according to the existing research results were as follows [43]: Figure 2, Figure 3 and Figure 4 presented the results of the SEM (Akishima, Tokyo, Japan), XRD (Bruker-AXS, Shibuya, Tokyo, Japan), and UV-Vis (Gangnam, Seoul, Korea) analysis for NST with optimum mix proportion. It was found from Figure 2 that there was only the peak of anatase phase with excellent photocatalysis. However, the peaks of the rutile and brookite phases were not observed, because SiO_2_ with relatively better thermal properties interrupted the phase transition during the heat treatment process. Results of the SEM analysis in Figure 3 showed no aggregation of NST or single phases of TiO_2_ or SiO_2_.

As shown in Figure 4, it was well known that TiO_2_ was activated in the wavelength range below 380 nm to act as a photocatalyst. The UV/Vis spectrophotometer testing was conducted to analyze the absorption spectra of NST. Test results showed that UV absorption peak of NST was found in the ultraviolet range below 380 nm, with much higher absorption than general TiO_2_.

#### 3.1.2. Cement

The cement used for this study was ordinary Portland cement (OPC) (Hanil, Seoul, Korea), which had a density of 3.14 g/cm^3^ and Blaine fineness of 3,492 cm^2^/g. The physical and chemical properties of OPC were as shown in Table 2.

#### 3.1.3. Silica Sand

The fine aggregate used in this study, silica sand (Hanil-Eco, Gongju, Korea), was used instead of general natural fine aggregate, and the physical properties of silica sand were shown in Table 3.

#### 3.1.4. Aggregate

The coarse aggregate used in this study was crushed stone (Hanil-Eco, Gongju, Korea). The grain size of the aggregate was 5 to 8 mm, and the physical properties of the coarse aggregate were shown in Table 4.

### 3.2. Production of Concrete Block

As a result of testing the mortar mixed with NST, the flow rate of the mortar decreased, and the strength increased as the NST replacement rate increased. The increase in the strength could be attributed to the pozzolan reaction of SiO_2_, the nucleation and filling effects of NST, and the formation of pores smaller than 50 nm within the mortar matrix. Accordingly, 4% was found to be the optimal amount of NST to mix in order to obtain the target strength and flow value without using excessive chemical admixture.

**Figure 5:**
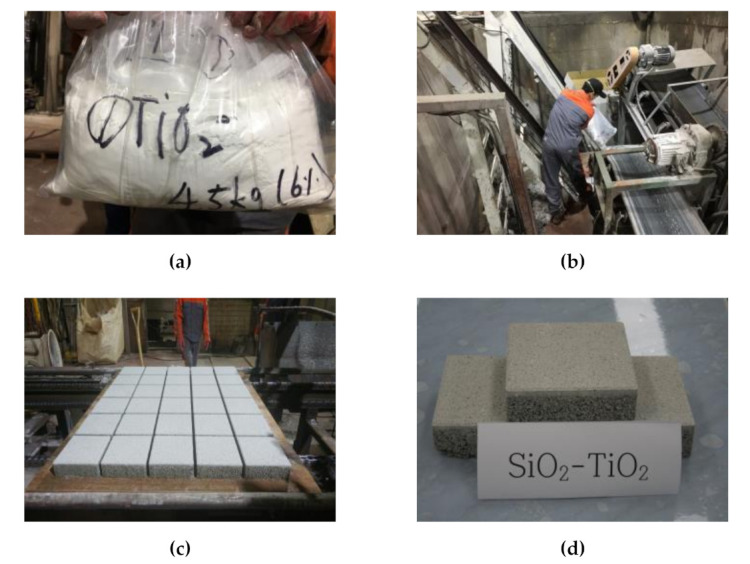
Manufacture of the concrete blocks: (**a**) material preparation; (**b**) manufacture; (**c**) block production; and (**d**) concrete block.

Table 5 showed the mix proportions of the concrete blocks prepared in this study. The concrete block consisted of surface and concrete layers. As for the surface layer of the concrete block, which was 8 mm high, 4% of NST was applied to the mixture. For the 52 mm concrete layer, 5–8 mm aggregates were used. As shown in Figure 5, they were made into a standard square of 200 × 200 × 60 mm using equipment for manufacturing concrete blocks.

### 3.3. Experimental Program

#### 3.3.1. Laboratory Experiment

**Figure 6:**
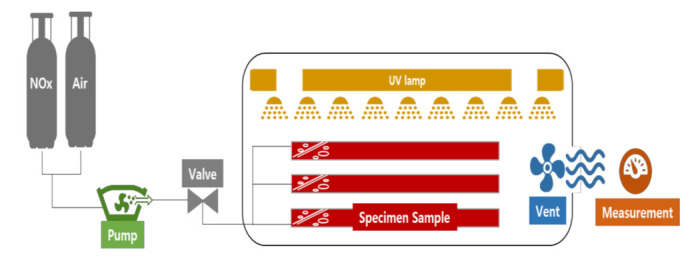
Schematic representation of the experimental setup.

**Figure 7:**
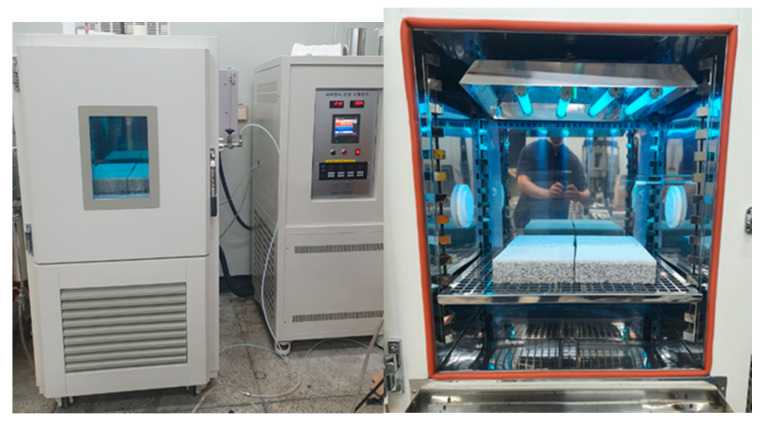
Laboratory experiment with environment chamber.

A laboratory experiment was conducted to assess the capacity of the concrete block with NST to remove the NO_x_ pollutants. The laboratory testing device (Ecotech, Knoxfield, Australia) with environment chamber was manufactured to evaluate the photocatalytic efficiency of concrete block specimens. The test setup was adapted from ISO 22197-1 (2007): “Test method for air-purification performance of semiconducting photocatalytic materials-Part1: Removal of Nitric Oxide” and UNI 11247: 2010: “Determination of the degradation of nitrogen oxides in the air by inorganic photocatalytic materials: continuous flow test method”. The developed experimental setup consisted of a pollutant source (gas cylinder of NO), zero air source, adjustable valves, humidifier, calibrator, photoreactor, and chemiluminescent NO_x_ analyzer, as shown in Figure 6 and Figure 7.

The calibrator controlled the NO_x_ concentration flowing into the environment chamber, and a pressure gauge and a valve were in place to control NO_x_ inflow. A T-shaped connector before the photoreactor was linked to the NO_x_ analyzer to measure the concentration in the inlet. A gas mixture of NO_x_ and zero air filled the photoreactor at the controlled humidity, flow, and NO_x_ concentration.

The chamber’s photoreactor (500 × 500 × 500 mm) was fully sealed to maintain the controlled environment. All tests were performed at a temperature of 25 ± 2 °C and a humidity level of 40 ± 5%. UV light was placed above the photoreactor to simulate for photocatalysis.

The concrete block was placed in the center of the photoreactor, and the concentration of NO_x_ was set to be 1.00 ppm by adding a gas mixture of NO_x_ and zero air before the testing. The light source of the UV lamp was irradiated under three conditions of 10, 20, and 30 W/m^2^ for more than five hours to measure changes in the NO_x_ concentration at one-minute intervals.

#### 3.3.2. Full-Scale Environment Chamber Experiment

**Figure 8:**
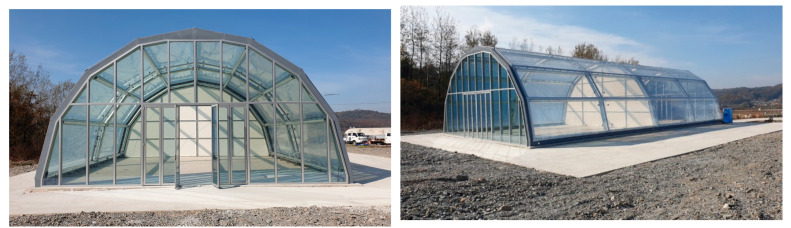

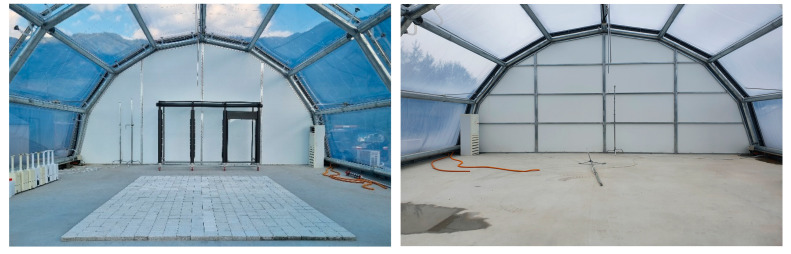
KICT Full-Scale Environment Chamber.

The full-scale environment chamber (KICT, Yeoncheon, Korea) had been constructed to evaluate the air pollutant reduction technologies on the roadside at the Korea Institute of Civil Engineering and Building Technology (KICT) near Yeoncheon, Korea (Figure 8). The chamber was a tunnel in shape, with a width of 11.4 m, a length of 22.8 m, and a height of 6.0 m. The total volume of this chamber was 1000 m^3^. This chamber was fabricated from transparent ETFE (Ethylene Tetrafluoroethylene) film to enable natural sunlight and ambient temperatures to govern the photochemical reactions occurring inside the chamber. The ETFE film was selected as the membrane material for the full-scale chamber structure because of its lightweight, high tensile strength, good durability, and high solar transmittance [44,45]. A previous study proved that ETFE films could supply sufficient natural sunlight for the photochemical reaction in the environment chambers, at an average of approximately 89% natural sunlight transmission at 300–1000 nm [46].

The environment chamber was divided into reference and testing chambers. The membrane layer was installed in the middle of chamber for perfectly blocking the transportation of aerosol and gas materials between the two chambers. The test data measured in the reference chamber was used to determine the wall loss rates and leakage rates of gaseous reactants and particles. It was applied to calibrate the measurement data in the testing chamber for the analysis. The temperature and humidity control systems were implemented at each chamber to maintain the designed environment condition. A total of 16 sensors at different heights were installed to monitor the temperature and relative humidity data inside the chamber during the testing.

The testing was conducted to determine NO/SO_2_ reduction efficiency of concrete blocks with TiO_2_ application using a full-scale environment chamber. The chamber was purged with rural background air prior to the experiment. The diesel and gasoline exhaust gas were generated from a 2010 Hyundai Star Rex (Hyundai, Ulsan, Korea) and 2019 Sonata engine (Hyundai, Ulsan, Korea), which were run under idling conditions. An air fan was used to uniformly diffuse the exhaust gas inside the chamber within a short period of time.

A total of 50 concrete blocks were placed at the center of the testing chamber with 4m^2^ of coverage area. The concrete blocks were exposed to natural sunlight for photocatalytic reaction by removing the aluminum cover after ensuring an equilibrium condition. The environment parameters such as temperature, relative humidity, and solar radiation were monitored during the testing at one-minute intervals.

## 4. Test result

### 4.1. Laboratory Experiment

**Figure 9:**
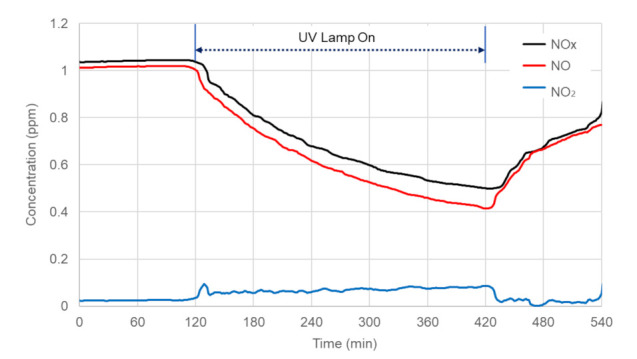
Variation of NO_x_ concentrations during the experiment (UV intensity = 30 W/m^2^).

Figure 9 illustrated the variation of NO_x_ concentration during the laboratory environment chamber test for the concrete block sample treated with 4% of NST replacement rate under 30W/m^2^ of UV intensity. The inlet concentration reached equilibrium at 1 ppm before the light was turned on. After turning on the UV light, a fast drop of NO and NO_x_ concentration in the outlet was exhibited, and the NO^2^ was produced from the NO oxidation. The light and gas supply were turned off after five hours of testing. For the test condition shown in Figure 9, the use of NST photocatalyst had a NO reduction of 59.1%, and the overall NO_x_ reduction was 51.8%.

**Figure 10:**
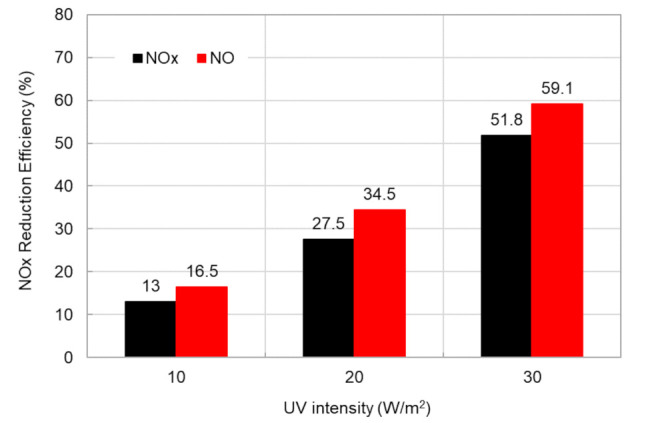
Effects of UV light intensity on NO_x_ reduction efficiency.

Figure 10 presented the effects of UV light intensity on NO_x_ reduction efficiency. Results presented in Figure 10 showed that the NO/NO_x_ reduction efficiency doubled with an increase of 10 W/m^2^ in UV intensity. As a result of the experiment, the concrete blocks using NST were capable of reducing the NO_x_ concentration through photocatalysis. This fact proved that NST does not penetrate the cement pores and was evenly dispersed in the surface layer of the concrete blocks, since negative charges were formed due to the Ti-O-Si bond and activated interface.

### 4.2. Full-Scale Environment Chamber Experiment

**Figure 11:**
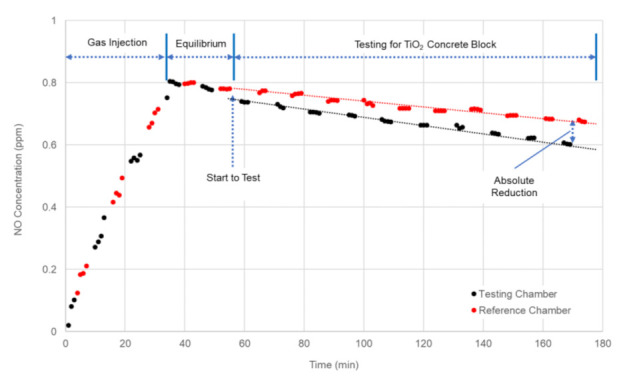
Variation of NO concentrations during the experiment.

Figure 11 illustrated the variation of NO concentration measured in reference and testing chambers during the full-scale experiment for the concrete block specimens treated with TiO_2_ material. As shown, the NO concentration rapidly increased in both chambers, by injecting the exhaust gas for roughly 40 min, until NO concentration inside the chamber reached approximately 800 ppb. After stopping the gas injection, it took 20 min to ensure equilibrium conditions inside the chambers. Once the concrete blocks were exposed to natural sunlight in the testing chamber, a fast drop of NO concentration was exhibited due to the photocatalytic reaction of TiO_2_ material. To determine NO/SO_2_ reduction efficiency of TiO_2_ concrete blocks in the environment chamber, two hours of measured data were compared between the reference and testing chambers. The measured data in the reference chamber was used to calculate the absolute reduction of NO/SO_2_ concentration by considering wall loss and leaking phenomenon. The testing was conducted at different weather conditions and times to investigate the effects of solar radiation on NO/SO_2_ reduction efficiency.

**Figure 12:**
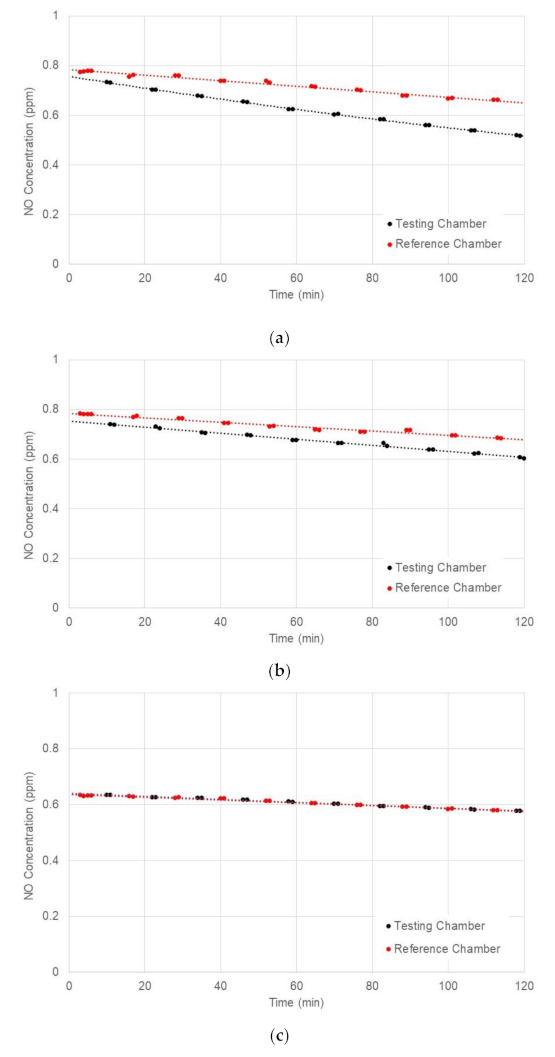
Comparison of NO concentrations measured in testing and reference chambers at different levels of solar radiation: (**a**) Average Solar Radiation = 564 W/m^2^; (**b**) Average Solar Radiation = 358 W/m^2^; and (**c**) Average Solar Radiation = 67 W/m^2^.

Figure 12 compared the nitrogen oxide (NO) concentration data measured from the reference and testing chambers for different levels of solar radiation. For the reference chamber, it was observed from test results that the NO concentration decreased linearly with time at 0.5~1 ppb/min.

As shown in Figure 12, the NO concentrations in the testing chamber were significantly reduced after the application of natural sunlight at high levels of solar radiation. The difference in NO concentration measured in the testing and reference chambers gradually increased with time due to the continuous photochemical reaction. However, there were no differences in NO concentration between the testing and reference chambers at very low solar radiation, indicating that the average 67 W/m^2^ of solar radiation was not enough for activating a photocatalytic reaction of concrete blocks with TiO_2_ application.

Table 6 presented the absolute reduction and reduction efficiency of NO measured after two hours in three different solar radiation levels. At high levels of solar radiation, the absolute reduction and reduction efficiency of NO were 0.145 ppm and 22.3%, respectively. It was found from this table that NO reduction efficiency tended to increase as the level of solar radiation increased.

**Figure 13:**
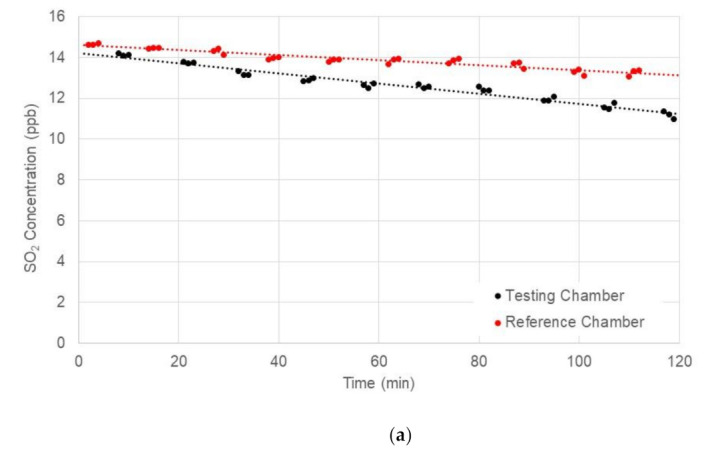

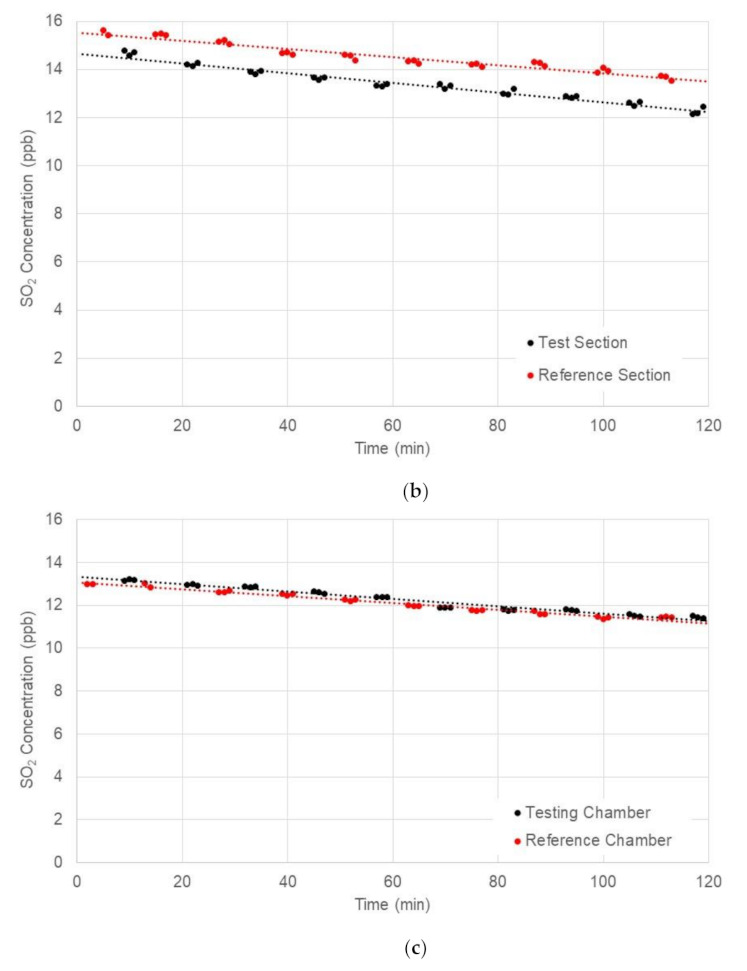
Comparison of SO_2_ concentrations measured in testing and reference chambers at different levels of solar radiation: (**a**) Average Solar Radiation = 564 W/m^2^; (**b**) Average Solar Radiation = 358 W/m^2^; and (**c**) Average Solar Radiation = 67 W/m^2^.

Figure 13 presented the comparison of SO_2_ concentrations measured in the testing and reference chambers. It was observed from this figure that the SO_2_ concentration in the testing chamber could be reduced using TiO_2_ at 358 and 564 W/m^2^ solar radiation. Similar to the NO measurement results, the SO_2_ reduction efficiency of concrete blocks with TiO_2_ applications tended to increase with an increase of solar radiation. It was also found from the test results that the reduction efficiencies of SO_2_ were slightly lower than those of NO at high solar radiation levels.

Table 7 presented the measured SO_2_ reduction efficiencies for the concrete block that was treated with TiO_2_. It was found that the use of TiO_2_ photocatalyst materials had an SO_2_ reduction of 14.4% at 564 W/m^2^ of average solar radiation.

## 5. Conclusions

This study evaluated the environmental effectiveness of incorporating NST as an additive to concrete blocks. The NST coated with SiO_2_ as a support was prepared using the sol-gel method and blended with a conventional concrete binder at 4% of replacement rate for concrete blocks. Prepared concrete block samples were evaluated for the NO_x_ reduction performance using laboratory and full-scale chamber experiments. Based on the results of the experimental programs, the following conclusions may be drawn:

(1) Based on the results of the laboratory chamber test, the concrete blocks using NST were effective in reducing NO_x_ pollutants in the air stream. The NO reduction efficiency ranged from 16.5 to 59.1%, and the highest NO reduction efficiency was achieved with a UV intensity of 30 W/m^2^. The increase in UV intensity positively affected the effectiveness of the NO_x_ reduction capacity.

(2) Results of the full-scale environmental chamber test showed that the concrete blocks with NST was effective in reducing NO and SO_2_ pollutants. The maximum environmental performance was achieved at a 564 W/m^2^ with NO and SO_2_ reduction efficiencies of 22.3% and 14.4%, respectively.

(3) In the future, the field application of NST as a road material is required to validate the NO_x_ and SO_2_ reduction efficiencies by considering various influencing parameters. In order to improve the photocatalysis efficiency of TiO_2_, the TiO_2_ material has been developed as an anatase crystal phase with larger specific surface area and excellent photodissociation. Various attempts have been made, including sputtering, a process in which TiO_2_ is directly and physically coated on a proper and solid support.

## Figures and Tables

**Table 1:** Physical properties of NST.

Density (g/cm^3^)	Surface Area (m^2^/g)	Particle Size (nm)	Pore Volume (cm^3^/g)	Pore Size (Å)	pH
2.4	337	6.3~11.5	0.31	52	6~6.5

**Table 2:** Physical and Chemical properties of OPC.

Density (g/cm^3^)	Blaine Fineness (cm^2^/g)	Chemical Properties (%)						
		SiO_2_	Al_2_O_3_	Fe_2_O_3_	CaO	MgO	SO_3_	Ig.loss
3.14	3,492	21.1	4.65	3.14	62.8	2.81	2.1	2.18

**Table 3:** Physical properties of silica sand.

Type	Density (g/cm^3^)	Diameter of Particle (mm)	Absorption (%)	SiO_2_ Content (%)
#4	2.61	0.8∼1.18	0.8	97.2

**Table 4:** Physical properties of aggregate.

Grading (mm)	Density (g/cm^3^)	Unit Weight (kg/m^3^)	Absorption (%)	Ratio of Absolute Volume (%)
5~8	2.75	1.510	0.84	52

**Table 5:** Mix proportion of concrete block.

Type	Grading of Agg.	W/B (%)	Thickness (mm)	Mix Proportioning (Ratio)		
				OPC	NST	Aggregate
Surface Layer	Silica sand No. 4	25	8	1	0.04	3
**Type**	**Grading** **of Agg.**	**W/B** **(%)**	**Target Void Ratio (%)**	**Unit Weight (kg/0.7m^3^)**		
				**OPC**	**Water**	**Aggregate**
Concrete Layer	5∼8 mm	25	6	413	95	1876

**Table 6:** Summary of NO Absolute Reduction and Reduction Efficiency.

Test ID	Average Solar Radiation (W/m^2^)	Average UV Intensity (W/m^2^)	NO Concentration (ppm)		Absolute Reduction (ppm)	Reduction Efficiency (%)
			Reference Chamber	Testing Chamber		
1	564	30.2	0.6508	0.5056	0.1452	22.3
2	358	19.8	0.5883	0.5247	0.0636	10.8
3	67	2.9	0.5802	0.5768	0.0034	0.6

**Table 7:** Summary of SO_2_ Absolute Reduction and Reduction Efficiency.

Test ID	Average Solar Radiation (W/m^2^)	Average UV Intensity (W/m^2^)	NO Concentration (ppm)		Absolute Reduction (ppm)	Reduction Efficiency (%)
			Reference Chamber	Testing Chamber		
1	564	30.2	13.106	11.22	1.886	14.4
2	358	19.8	11.668	11.155	0.513	10.0
3	67	2.9	11.145	11.259	−0.114	−1.0

## Data Availability

Data sharing is not applicable.
